# Practical Departments

**Published:** 1895-07-06

**Authors:** 


### PRACTICAL DEPARTMENTS.

### THE PATENT "SIMMONS GIG."

Messrs. Simmons and Co., the well-known makers of children's carriages, perambulators, &c., of Tanner Street, S.E., have brought to our notice their very large stock of every sort and description of invalid chairs, spinal carriages, and more ordinary children's conveyances, for a speciality in which they have deserved reputation. Especially we must commend the patent "Simmons gig," which is a most convenient and delightful little carriage for small people. It can be used in a variety of ways, by ingenious methods of folding and detaching, so that whether as a spinal carriage or ordinary chair it is equally well arranged. The bed, for use in the former case, folds together and can be stowed away beneath the seat. The hood, which is like that of a hansom cab, will put entirely or half way down, and when up serves as efficient protection from sun or rain. There are curtains, which are withdrawable, also attached. The sides are detachable, and the shafts are folding. The gig is made in wood or wicker, in two qualities, the price varying from 60s. to 105s. 9d. There are various extras in the way of rubber and pneumatic tyres which can be added where desired. Altogether it would be difficult to imagine a more perfect little carriage of its kind than the one in question. We should not omit to mention that the gig is also made in adult size, price 300s.

